# Early-admission serum albumin and hospitalization burden in a selected serial-culture cohort of patients with diabetic foot ulcers and subsequent *Pseudomonas aeruginosa* detection: a retrospective cohort study

**DOI:** 10.3389/fendo.2026.1918069

**Published:** 2026-10-01

**Authors:** Feifei Kan, Aihong Wang, Youliang Tan, Huizhen Dai

**Affiliations:** Affiliated Hospital of Shandong Second Medical University, Weifang, Shandong, China

**Keywords:** diabetic foot ulcer, hospital charges, length of stay, prognostic assessment, *Pseudomonas aeruginosa*, serum albumin

## Abstract

**Background:**

Patients with diabetic foot ulcers (DFUs) who undergo repeat wound culture and subsequent *Pseudomonas aeruginosa* (PA) detection form a selected clinical trajectory. Early-admission serum albumin reflects inflammation, capillary permeability, hepatic synthesis, protein loss, hydration, and nutritional reserve; it is not a stand-alone measure of nutritional status. We examined its association with hospitalization burden conditional on this serial-culture trajectory.

**Methods:**

This single-center retrospective cohort included 408 hospitalized patients with DFU who had a PA-positive wound culture after an earlier non-PA culture during the same admission. Because cohort eligibility required continued hospitalization until repeat sampling and later PA detection, inference was conditional on a post-admission selection event susceptible to immortal-time and collider-selection bias. The culture sequence was an operational ascertainment pattern, not evidence of nosocomial acquisition, microbiological conversion, or invasive PA infection. The co-primary outcomes were length of stay (LOS) and institution-specific nominal hospital charges. Negative binomial and gamma log-link models estimated adjusted within-cohort associations. Exploratory prognostic analyses used bootstrap optimism correction and same-center temporal assessment (development, 2013–2016; assessment, 2017–2018).

**Results:**

Among 408 patients [261 men (64.0%)], median LOS was 14 days [interquartile range (IQR), 9–19] and median nominal hospital charges were CNY 18,708 (IQR, 12,532–27,979). Compared with albumin ≥35 g/L, albumin 30 to <35 g/L was associated with longer LOS [adjusted rate ratio (aRR), 1.24; 95% CI, 1.10–1.40] and higher charges [adjusted charge ratio (aCR), 1.28; 95% CI, 1.12–1.46]; corresponding estimates for albumin <30 g/L were 1.52 (95% CI, 1.29–1.78) and 1.67 (95% CI, 1.40–1.99). Per 5-g/L lower albumin, the aRR for LOS was 1.19 (95% CI, 1.14–1.25) and the aCR for charges was 1.22 (95% CI, 1.16–1.28). Baseline-only estimates changed by less than 3% on the log-ratio scale. Adding albumin increased the apparent area under the curve (AUC) from 0.679 to 0.758 (optimism-corrected, 0.643 to 0.729; ΔAUC, 0.079; bootstrap 95% CI, 0.036–0.120). In the same-center temporal assessment sample (126 patients; 50 events), the AUC was 0.592 for the core model and 0.719 after albumin was added; corresponding Brier scores were 0.234 and 0.204, and calibration slopes were 0.625 and 0.994. These small-sample performance estimates were treated as descriptive. The median bootstrap-optimal albumin cutoff was 35 g/L (95% bootstrap percentile interval, 33–37 g/L).

**Conclusions:**

Within this selected serial-culture cohort, lower early-admission albumin was associated with greater hospitalization burden and with numerically better exploratory prognostic performance in same-center analyses. These findings do not establish causality, transportability, a diagnosis of malnutrition, or a clinically actionable albumin threshold.

## Graphical abstract

**Figure:**
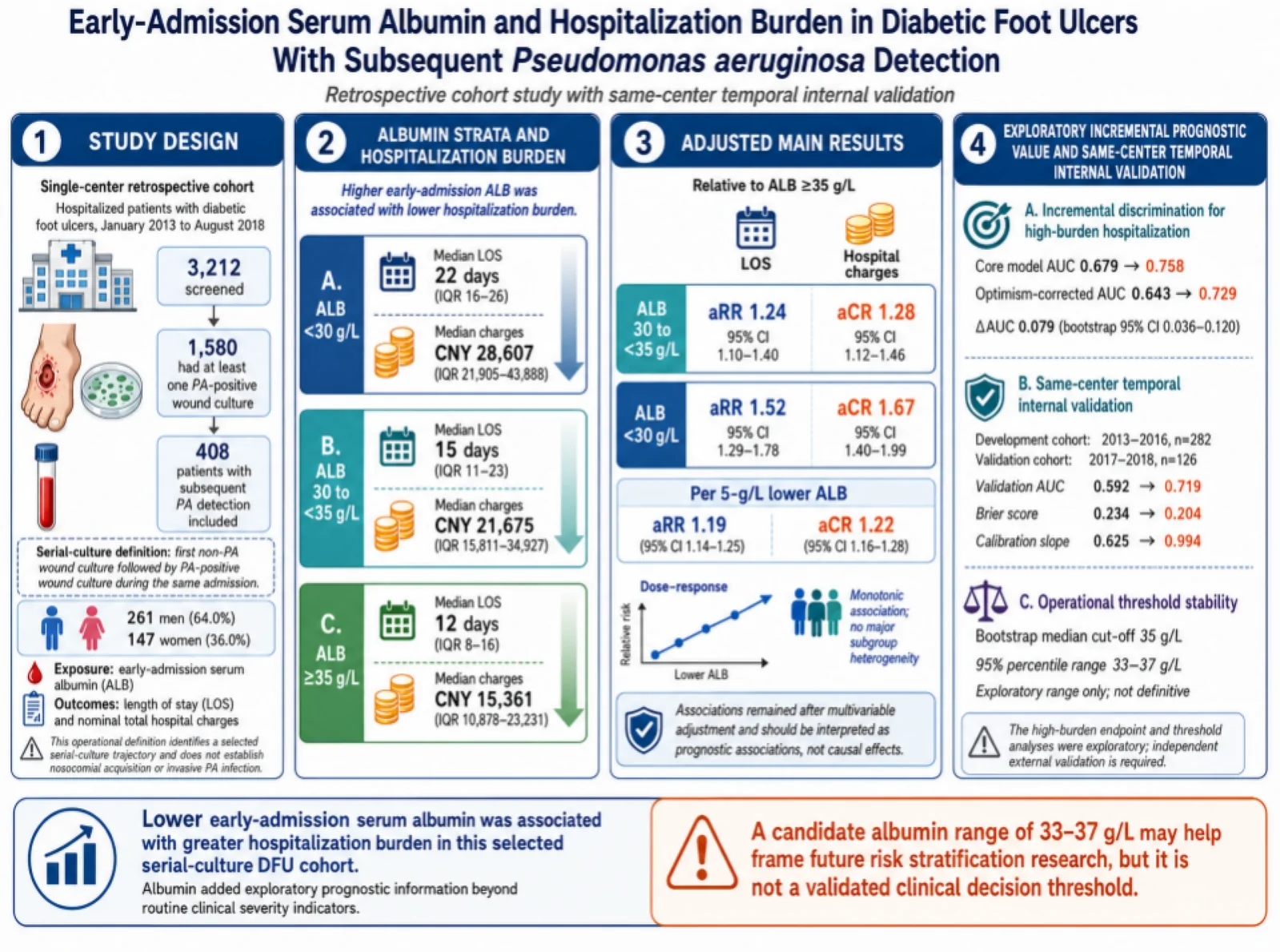
Early-admission serum albumin and hospitalization burden within a selected serial-culture cohort of patients with DFU and subsequent PA detection after an initial non-PA culture. Lower albumin was associated with progressively longer LOS and higher nominal hospital charges. Prognostic and threshold analyses were exploratory, restricted to the source center and healthcare system, and require independent external validation. ALB, serum albumin; aCR, adjusted charge ratio; aRR, adjusted rate ratio; AUC, area under the receiver operating characteristic curve; DFU, diabetic foot ulcer; LOS, length of stay; PA, *Pseudomonas aeruginosa*.

## 1 Introduction

Diabetic foot ulcers (DFUs) are a common and disabling complication of diabetes and a major cause of hospitalization, lower-extremity amputation, impaired quality of life, and healthcare expenditure (1–4). International guidance emphasizes integrated assessment of ulcer depth, ischemia, clinical infection severity, and comorbidity rather than reliance on culture results alone (5–7).

*Pseudomonas aeruginosa* (PA) is recovered from a variable proportion of diabetic foot specimens across regions and clinical settings (8–10). This variability reflects sampling technique, prior antibiotic exposure, wound chronicity, local microbial ecology, and the distinction between colonization and clinically active infection (10–12). Patients with PA detected in the initial admission specimen and those with PA detected only after repeat assessment follow different care pathways. In this study, later recovery after an earlier non-PA specimen was treated as a serial-culture trajectory that could reflect persistent or worsening findings, peri-debridement or intraoperative sampling, or reassessment of an unsatisfactory clinical course.

This sequence is an ascertainment pattern, not evidence of nosocomial acquisition, microbiological conversion, progression from colonization to invasive infection, or PA causation. Eligibility also required continued hospitalization and an opportunity for repeat sampling, creating immortal-time and collider-selection concerns (13). We therefore estimated the association between early-admission albumin and hospitalization burden conditional on this selected trajectory, rather than the effect of subsequent PA detection itself.

Serum albumin is routinely measured early during hospitalization and reflects the combined influence of inflammation, capillary permeability, hepatic synthesis, protein loss, hydration, and nutritional reserve. The American Society for Parenteral and Enteral Nutrition (ASPEN) advises that albumin and prealbumin primarily characterize inflammatory risk rather than nutritional status or total body protein, and the Global Leadership Initiative on Malnutrition (GLIM) requires phenotypic and etiologic criteria rather than albumin alone for the diagnosis of malnutrition (14, 15). Lower albumin has been associated with impaired wound healing, amputation, and adverse outcomes in DFU populations (16–21), but it should be interpreted here as a nonspecific illness-risk marker rather than a validated nutritional diagnosis or modifiable treatment target.

We evaluated the association of early-admission serum albumin with length of stay (LOS) and nominal hospital charges among patients with DFU and subsequent PA detection after an initial non-PA culture. The primary aim was to characterize the adjusted within-cohort association and dose–response pattern. Prespecified and exploratory subgroup analyses assessed effect heterogeneity. A secondary, explicitly exploratory aim was to assess whether albumin added prognostic information beyond a parsimonious clinical model. Flexible modeling, optimism correction, calibration assessment, and same-center temporal assessment within the source system were used to limit overinterpretation of apparent performance (22–26).

## 2 Methods

### 2.1 Study design and setting

This retrospective cohort study was conducted at the Affiliated Hospital of Shandong Second Medical University and included consecutive patients hospitalized with DFU between 1 January 2013 and 31 August 2018. Reporting followed the Strengthening the Reporting of Observational Studies in Epidemiology (STROBE) statement; the exploratory prognostic component was reported with reference to Transparent Reporting of a Multivariable Prediction Model for Individual Prognosis or Diagnosis (TRIPOD) reporting principles and Prediction Model Risk of Bias Assessment Tool (PROBAST) concepts (23, 26, 27). The institutional ethics committee approved protocol SR201301002 in 2013 for secondary analysis of de-identified routinely collected clinical data and determined that no additional written informed consent was required. The present study has also been approved by protocol protocol SDSMU-2026-ky-287.

### 2.2 Participants and operational definition of subsequent PA detection

Eligible patients were identified from the electronic medical record. Patients with at least one PA-positive wound culture formed the source PA-positive-culture cohort. Subsequent PA detection was defined as recovery of PA from a wound specimen obtained after an earlier specimen from the same index hospitalization in which PA had not been recovered. Because eligibility was determined only after the later PA-positive specimen, every included patient had to remain hospitalized and undergo repeat sampling; patients without repeat sampling or with a second negative culture were not included. This operational sequence describes chronological microbiological detection and a clinical-reassessment trajectory; it does not establish nosocomial acquisition, true microbiological conversion, progression from colonization to invasive infection, or a causal role of PA in resource use. The estimand was the association of early-admission albumin with outcomes conditional on this selection, not the effect of PA detection.

Diabetes-related foot infection and its severity were assessed independently of culture findings using the International Working Group on the Diabetic Foot/Infectious Diseases Society of America (IWGDF/IDSA) classification. For this analysis, PA was treated as a detected organism rather than an adjudicated causative pathogen. Contemporary IWGDF/IDSA guidance favors aseptically collected tissue after cleansing and debridement when culture is indicated and cautions that repeat cultures in nonresponding wounds may recover resistant contaminants rather than true pathogens (5).

Dates and times of hospital admission, collection of the initial non-PA specimen, and collection of the first PA-positive specimen were abstracted from electronic microbiology records. Culture intervals were calculated from specimen-collection dates rather than laboratory-report dates.

Repeat cultures were not mandated by a standardized surveillance protocol and were not systematically obtained in all patients with DFU. The documented indication for repeat sampling was classified as persistent or worsening local infection findings, peri-debridement or intraoperative microbiological reassessment, another documented clinical indication, or an indication that could not be reliably reconstructed from the available records.

Specimen types were classified as wound-secretion or superficial specimens, deep-tissue or curettage specimens, and intraoperative specimens. Specimen type was recorded separately for the initial non-PA and first PA-positive cultures. Because sampling depth and specimen quality may influence organism recovery, specimen type was treated as an ascertainment characteristic rather than evidence of microbiological acquisition or invasive PA infection.

Clinical eligibility required diabetes with a confirmed DFU and the prespecified serial non-PA-to-PA culture pattern. Exclusion criteria were non-DFU wounds, pre-admission systemic antibiotic exposure considered likely to compromise interpretation of the initial culture, active malignancy, end-stage hepatic or renal disease, and immunosuppression or immunodeficiency. In-hospital antibiotic exposure before PA detection was recorded because it could influence subsequent culture results. All 408 patients who met the clinical and serial-culture eligibility criteria had an early-admission albumin measurement, complete co-primary outcome and modeled-covariate data, and a complete discharge record; therefore, no patient was excluded because of missing analytic data.

During the study period, DFU management was clinician-directed and could include debridement, vascular assessment and revascularization, negative-pressure wound therapy, and antimicrobial treatment according to clinical findings. The database captured the listed treatments but did not document whether every patient was managed through a standardized multidisciplinary foot pathway or received formal nutritional consultation. Treatment variables therefore describe observed management rather than protocolized interventions.

### 2.3 Exposure: early-admission serum albumin

Early-admission serum albumin was defined as the first available serum albumin measurement after hospital admission and was measured by the central laboratory under routine quality-assurance procedures. Most admission-albumin studies use an early value, often within 24 h of admission (28). Because some measurements occurred after limited early treatment or beyond 24 h, the exposure is termed early-admission rather than strictly pre-intervention albumin. Albumin was analyzed continuously, using clinically familiar categories of <30, 30 to <35, and ≥35 g/L. Continuous and spline analyses were prioritized to avoid assuming a discrete biological threshold.

### 2.4 Covariates

Covariates were selected on clinical grounds. Baseline or early-admission variables included age, sex, diabetes duration, DFU duration, glycated hemoglobin (HbA1c), estimated glomerular filtration rate (eGFR), white blood cell count (WBC), C-reactive protein (CRP), Wagner grade, IWGDF/IDSA infection grade, osteomyelitis, smoking status, Charlson Comorbidity Index, and admission year. Management or subsequently ascertained variables included lower-limb revascularization, negative-pressure wound therapy, antipseudomonal antimicrobial coverage, in-hospital antibiotic exposure before PA detection, and multidrug-resistant (MDR) PA phenotype. Serum creatinine-derived eGFR was retained to represent kidney filtration function. Urine albumin-to-creatinine ratio (UACR) could not be calculated from the available serum albumin and serum creatinine because UACR requires urine albumin and urine creatinine measured in the same urine specimen (40). After re-review of the analytic variables, neither urine albumin nor a paired urine creatinine measurement was available, and 24-h urinary protein had not been quantified. Thus, the models adjusted for measured filtration function but not renal protein loss. WBC and CRP were included as the available inflammatory covariates. Neutrophil-to-lymphocyte ratio, platelet-to-lymphocyte ratio, systemic immune-inflammation index, and related indices could not be derived because differential leukocyte and platelet measurements were not sufficiently complete. A CRP-to-albumin ratio was not entered as an adjustment covariate because it mathematically incorporates the exposure of interest. Other hepatic synthetic-function markers and validated nutritional or frailty measures were also not sufficiently complete for analysis. Management-extended models included both sets of variables; baseline-only sensitivity models excluded the five management or subsequently ascertained variables. Wagner grade, IWGDF/IDSA grade, smoking status, and admission year were entered as factors in categorical-albumin models, whereas ordered grade scores and a centered linear admission-year term were used in continuous and spline models. Alternative factor coding of the continuous models produced materially similar albumin estimates. HbA1c was prespecified to represent antecedent glycemic exposure. Fasting plasma glucose (FPG) was retained as a descriptive admission variable because a single value during acute hospitalization may reflect short-term stress and treatment-related glycemic variation rather than longer-term glycemic control.

Wagner and IWGDF/IDSA grades were assigned retrospectively and independently by two senior clinicians, with disagreements adjudicated by a third reviewer. Agreement was summarized using exact agreement and linearly weighted Cohen's κ with 95% CIs (29–31). Because several management variables occurred after admission and may lie on the pathway between baseline illness and hospitalization burden, estimates from the management-extended models were interpreted as management-conditional prognostic associations rather than causal effects, and baseline-only models were used to assess potential overadjustment (32).

### 2.5 Outcomes

Co-primary outcomes were LOS in days and total hospitalization charges in Chinese yuan (CNY), obtained from the hospital case-management system. The monetary outcome represents institution-specific inpatient charges rather than resource-based direct medical costs, reimbursement, patient out-of-pocket expenditure, or societal costs. Nominal charges were used in the primary analysis. In a sensitivity analysis, charges were standardized to 2018 CNY using annual national all-items consumer price inflation from the World Bank World Development Indicators; multipliers for admissions in 2013 through 2018 were 1.0935, 1.0729, 1.0577, 1.0369, 1.0207, and 1.0000, respectively (33). CPI adjustment corrects general inflation only and does not harmonize medical-service tariffs, treatment regimens, procedure mix, or institution-specific accounting. Admission year was represented as a six-level factor (five indicator terms, with 2013 as the reference) in categorical-albumin models and as a centered linear term in continuous and spline models. For exploratory threshold and decision-analytic analyses, high hospitalization burden was defined as LOS or charges at or above the sex-specific 75th percentile. This data-derived endpoint was not a consensus clinical definition and was treated as hypothesis-generating rather than validated. Post-PA LOS was defined as the number of days from collection of the first PA-positive specimen to discharge and was examined as a sensitivity outcome to reduce, but not eliminate, circularity arising because total LOS partly precedes cohort ascertainment. Total charges accrued over the entire admission and likewise included expenditures incurred before the qualifying PA-positive culture.

### 2.6 Statistical analysis

All analyses were performed in Python using NumPy, SciPy, statsmodels, Patsy, scikit-learn, and Matplotlib; exact software versions were documented in the analytic code record available from the corresponding author on reasonable request. The study size was determined by all eligible records during the study period; no *a priori* power calculation was used. Continuous variables were summarized as mean (SD) or median [interquartile range (IQR)], using one prespecified summary format for each variable across albumin groups; categorical variables were summarized as number (percentage). The final analytic dataset contained no missing values in variables used for the reported analyses, and no imputation was required. Group comparisons used one-way analysis of variance or the Kruskal–Wallis test for continuous variables and the χ² or Fisher exact test for categorical variables, as appropriate. Dunn *post-hoc* comparisons used Holm correction. Tests were two-sided with α = 0.05. Because LOS and charges were co-primary outcomes and subgroup, threshold, and prediction analyses were exploratory, effect estimates and 95% CIs were emphasized; no global multiplicity adjustment was applied beyond the specified *post-hoc* procedure.

Adjusted associations were estimated using negative binomial regression for LOS and gamma regression with a log link for charges. Management-extended models included all covariates listed in Section 2.4; baseline-only models omitted revascularization, negative-pressure wound therapy, antipseudomonal antimicrobial coverage, prior in-hospital antibiotic exposure, and MDR phenotype. Albumin was modeled categorically and continuously, with continuous effects reported per 5-g/L lower albumin. Results are expressed as adjusted rate ratios (aRRs; ratios of expected LOS) and adjusted charge ratios (aCRs). Changes between baseline-only and management-extended estimates were quantified on the log-ratio scale using unrounded model coefficients. Multicollinearity was evaluated using variance inflation factors (VIFs) for one-degree-of-freedom terms and generalized VIFs adjusted for degrees of freedom [GVIF_(1/(2 × df))_] for multilevel factors. Influence diagnostics included Cook’s distance and albumin-term DFBETAs; a sensitivity analysis excluded observations with an absolute albumin DFBETA greater than 2/√*N*. An analogous baseline-only negative binomial model was fitted for post-PA LOS. FPG was added to the management-extended LOS and charge models in a separate sensitivity analysis.

Restricted cubic splines with knots at the 5th, 35th, 65th, and 95th percentiles characterized the functional form of the albumin–outcome associations. Departure from linearity was evaluated using likelihood-ratio tests. Sex was prespecified as a potential effect modifier. Additional exploratory interactions examined Wagner grade (0–3 vs. 4–5), infection grade (0–2 vs. 3), CRP (<10 vs. ≥10 mg/L), MDR PA, prior antibiotic exposure, osteomyelitis, and admission period. Interaction terms, rather than within-stratum significance, were used to evaluate heterogeneity.

### 2.7 Exploratory prognostic assessment and same-center temporal assessment

Incremental prognostic value was assessed for the exploratory high-burden endpoint. A prespecified core logistic model included age, sex, Wagner grade, IWGDF/IDSA infection grade, log-transformed CRP, WBC, eGFR, Charlson Comorbidity Index, osteomyelitis, and admission year. The core model was compared with the same model plus continuous albumin. Apparent area under the curve (AUC), Brier score, and Akaike information criterion were reported. Optimism in AUC and Brier score was estimated using 1,000 bootstrap resamples, and a paired bootstrap distribution was used to obtain the 95% CI for ΔAUC.

For same-center temporal assessment, admissions from 2013 to 2016 formed the development sample and admissions from 2017 to 2018 formed the assessment sample within the same center, electronic record system, laboratory, and clinical service. Sex-specific 75th-percentile thresholds for LOS and charges were estimated only in the development sample and then frozen for assessment. Admission year was omitted from temporal models to avoid extrapolating a calendar-time coefficient. Assessment performance included AUC, Brier score, calibration intercept and slope, and prediction error for continuous LOS and charges [mean absolute error (MAE) and root mean squared error (RMSE)]. The assessment sample contained 126 patients and 50 high-burden events; its performance estimates, especially the calibration slope, were therefore considered imprecise and descriptive. The Youden-optimal albumin cutoff was recalculated in 2,000 full-cohort bootstrap samples to quantify threshold instability. Apparent discrimination, calibration, and decision-curve analyses for albumin alone were descriptive; because no albumin-triggered intervention was specified or tested, decision curves were not interpreted as evidence of clinical utility (34, 35). Consistent with TRIPOD and PROBAST principles, these analyses represent same-center temporal assessment within the source system, not external validation or proof of clinical usefulness (23–26).

## 3 Results

### 3.1 Cohort characteristics

Between January 2013 and August 2018, 3,212 patients hospitalized with DFU were screened. Of these, 1,632 had no PA-positive wound culture and 1,580 had at least one PA-positive wound culture. Among the latter, 1,172 did not meet the prespecified analytic eligibility criteria and 408 were included (**Figure 1**). The cohort comprised 261 men (64.0%) and 147 women (36.0%), with a mean age of 61.5 (SD, 10.1) years. Albumin was measured a median of 13 h (IQR, 8–19) after admission; 381 measurements (93.4%) occurred within 24 h and 404 (99.0%) occurred within 48 h. Before sampling, 108 patients (26.5%) received intravenous crystalloid, with a median volume of 500 mL (IQR, 250–750); no patient received human albumin before the index measurement.

**Figure 1:**
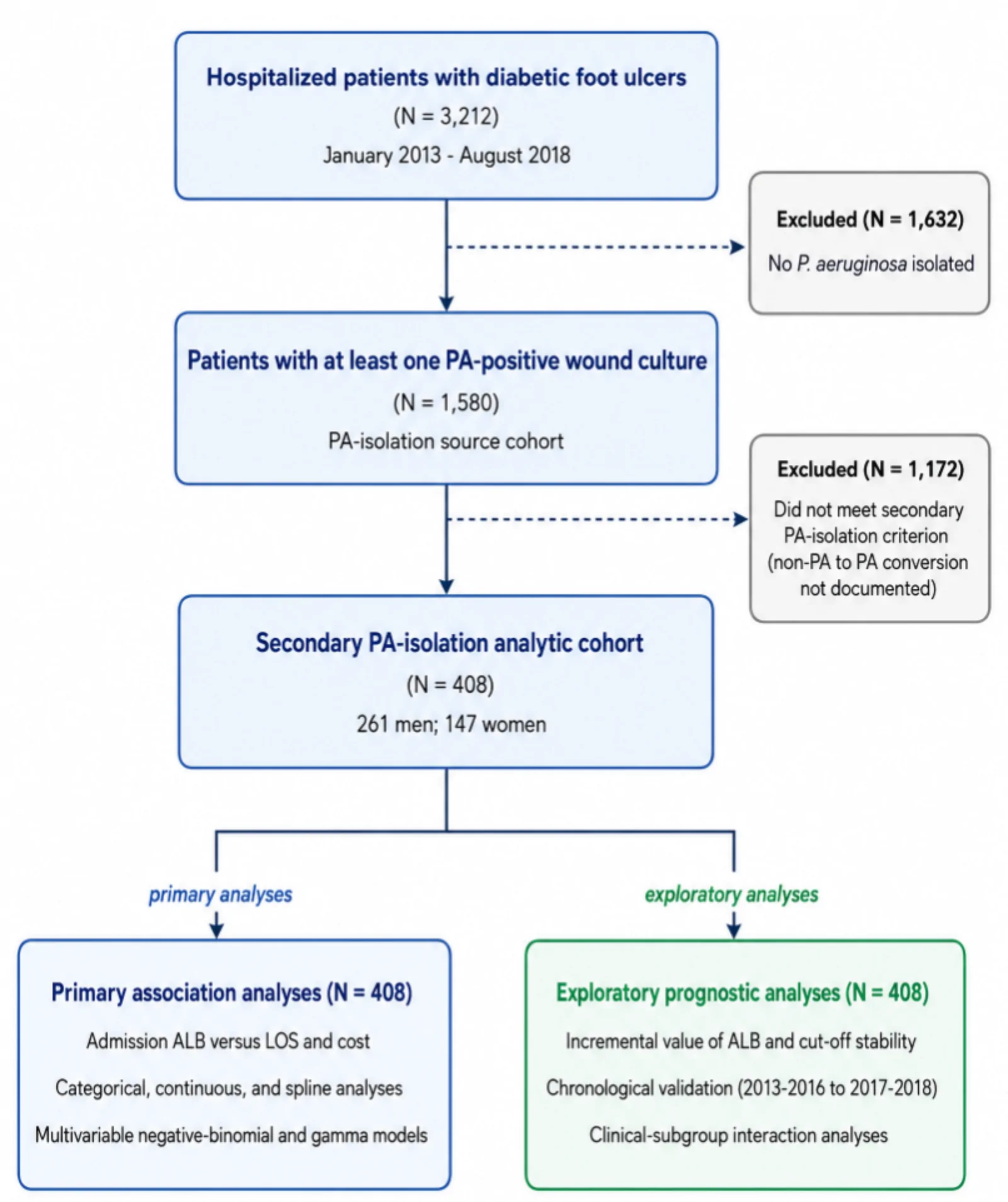
Study flow diagram. Of 3,212 hospitalized patients with DFU, 1,632 had no PA-positive wound culture and 1,580 had at least one PA-positive wound culture. Among the latter, 1,172 did not meet the prespecified analytic eligibility criteria and 408 were included. The later PA-positive culture defines a serial-detection trajectory but does not distinguish colonization from clinical infection or establish nosocomial acquisition. Because repeat sampling was required, the analytic cohort is conditioned on continued hospitalization and opportunity for sampling. DFU, diabetic foot ulcer; LOS, length of stay; PA, *Pseudomonas aeruginosa*.

MDR PA was identified in 35.3% of patients and osteomyelitis was identified in 20.8%. Median LOS was 14 days (IQR, 9–19), median nominal hospital charges were CNY 18,708 (IQR, 12,532–27,979), and median early-admission albumin was 36 g/L (IQR, 32–40; range, 18–50). The 5th and 95th percentiles were 26 and 45 g/L, and the 1st and 99th percentiles were 21.1 and 49.9 g/L, respectively. Fifty-six patients (13.7%) had albumin <30 g/L, 101 (24.8%) had albumin 30 to <35 g/L, and 251 (61.5%) had albumin ≥35 g/L. No otherwise eligible patient was excluded because of missing key analytic data.

### 3.2 Culture chronology, indications for repeat sampling, and specimen types

The initial specimen in which PA was not recovered was collected a median of 1 day (IQR, 0–2) after admission. The median interval from the initial non-PA specimen to the first PA-positive specimen was 6 days (IQR, 3–10), the median admission-to-PA interval was 7 days (IQR, 4–11), and median post-PA LOS was 7 days (IQR, 4–12).

Repeat sampling was not protocol-scheduled. A documented indication was available for 383 patients (93.9%): persistent or worsening local infection findings in 253 (62.0%), peri-debridement or intraoperative reassessment in 86 (21.1%), and another documented clinical indication in 44 (10.8%). The indication could not be reliably reconstructed in 25 patients (6.1%).

For the initial non-PA culture, 259 specimens (63.5%) were wound-secretion or superficial specimens, 102 (25.0%) were deep-tissue or curettage specimens, and 47 (11.5%) were intraoperative specimens. For the first PA-positive culture, the corresponding numbers were 221 (54.2%), 120 (29.4%), and 67 (16.4%), respectively.

### 3.3 Albumin strata and unadjusted hospitalization burden

Hospitalization burden decreased across increasing albumin strata (**Table 1**, **Figure 2**). Median LOS was 22 days (IQR, 16–26) in the <30-g/L group, 15 days (IQR, 11–23) in the 30-to-<35-g/L group, and 12 days (IQR, 8–16) in the ≥35-g/L group. Median nominal charges decreased from CNY 28,607 (IQR, 21,905–43,888) to CNY 21,675 (IQR, 15,811–34,927) and CNY 15,361 (IQR, 10,878–23,231), respectively (*p* < 0.001 for both outcomes). WBC was higher at lower albumin (*p* < 0.001), whereas age, diabetes and DFU duration, HbA1c, FPG, eGFR, and CRP (*p* = 0.204) did not differ across albumin strata.

**Table 1:** Selected baseline characteristics and outcomes by early-admission serum albumin stratum (*N* = 408).

Variable	Albumin <30 g/L (*n* = 56)	Albumin 30 to <35 g/L (*n* = 101)	Albumin ≥35 g/L (*n* = 251)	Overall (*N* = 408)	*p*-value
Male, *n* (%)	39 (69.6)	61 (60.4)	161 (64.1)	261 (64.0)	0.510
Age, years	61.9 ± 9.8	61.0 ± 10.6	61.5 ± 10.0	61.5 ± 10.1	0.872
Diabetes duration, months	128.3 ± 70.7	116.6 ± 67.2	114.4 ± 62.6	116.8 ± 64.9	0.349
DFU duration, months	2 (1–3)	2 (1–3)	2 (1–3)	2 (1–3)	0.582
HbA1c, %	8.3 ± 1.6	8.0 ± 1.7	7.8 ± 1.8	7.9 ± 1.7	0.090
FPG, mmol/L	7.9 ± 2.5	7.9 ± 1.9	7.4 ± 2.0	7.6 ± 2.1	0.087
eGFR, mL/min/1.73 m²	79.2 ± 26.3	82.0 ± 24.4	79.3 ± 25.0	80.0 ± 25.0	0.657
WBC, ×10^9^/L	9.5 ± 2.6	8.6 ± 2.3	8.0 ± 2.5	8.4 ± 2.5	<0.001
CRP, mg/L	17 (10–26)	15 (8–26)	13 (8–26)	14 (8–26)	0.204
Serum albumin, g/L	26.4 ± 2.9	32.4 ± 1.5	39.5 ± 3.8	36.0 ± 5.9	<0.001
LOS, days	22 (16–26)	15 (11–23)	12 (8–16)	14 (9–19)	<0.001
Nominal hospital charges, CNY	28,607 (21,905–43,888)	21,675 (15,811–34,927)	15,361 (10,878–23,231)	18,708 (12,532–27,979)	<0.001

Continuous variables are presented as mean ± SD or median (IQR), using the same summary format across all columns. *p*-values are from one-way analysis of variance or the Kruskal–Wallis test, as appropriate, and the χ² test for sex. CRP, C-reactive protein; DFU, diabetic foot ulcer; eGFR, estimated glomerular filtration rate; FPG, fasting plasma glucose; HbA1c, glycated hemoglobin; LOS, length of stay; WBC, white blood cell count.

**Figure 2:**
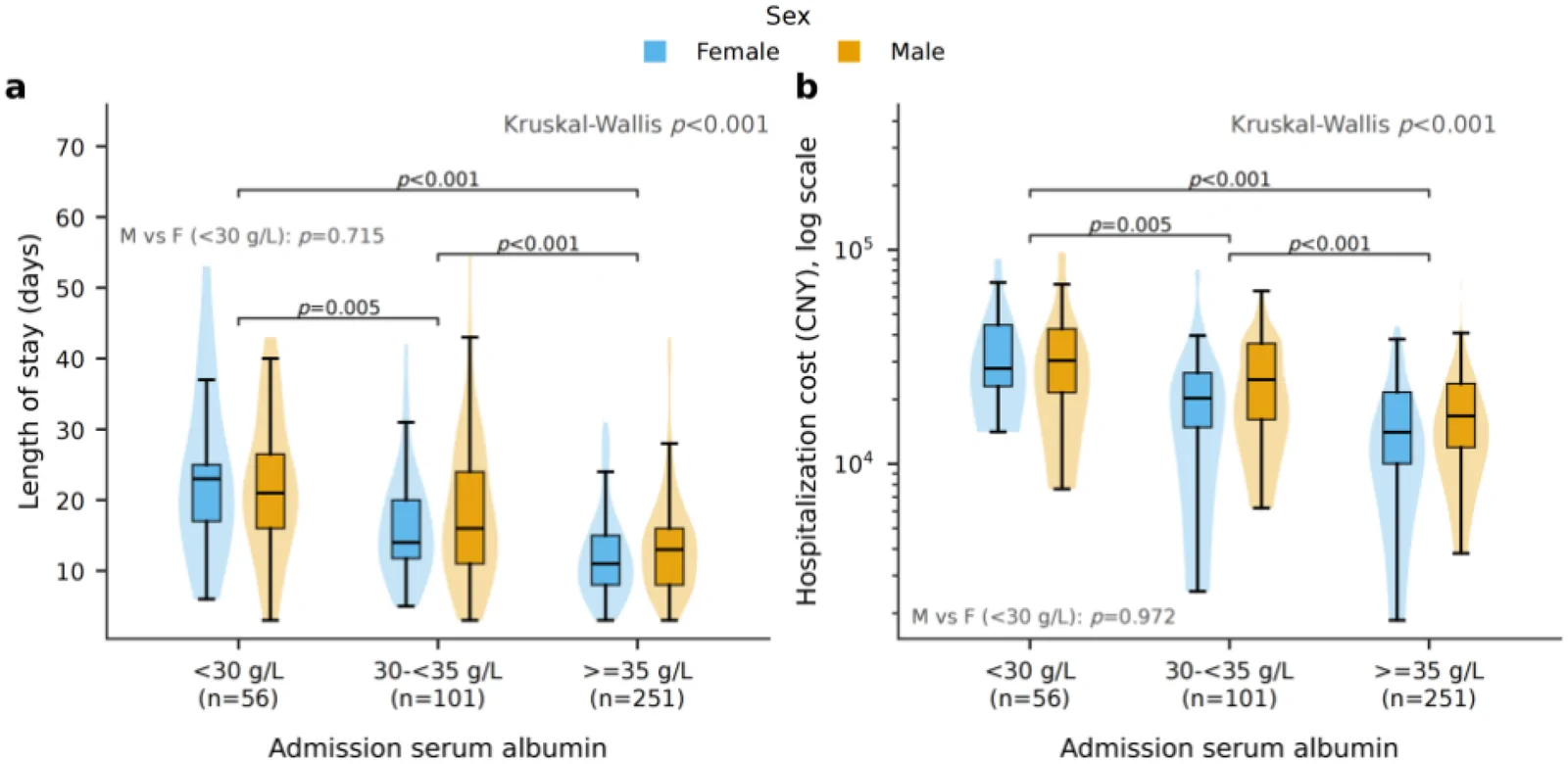
Length of stay **(a)** and nominal hospital charges [**(b)**; logarithmic scale] according to early-admission albumin category and sex. Violins show the distributions and boxes show medians and interquartile ranges. Overall *p*-values are from Kruskal–Wallis tests. CNY, Chinese yuan.

### 3.4 Descriptive findings across ulcer- and infection-severity strata

Patients with more severe ulcers or clinical infection had lower albumin and greater hospitalization burden (**Table 2**). Compared with Wagner grades 0–3, Wagner grades 4–5 were associated with lower albumin, longer LOS, and higher nominal charges; analogous differences were observed for IWGDF/IDSA grade 3 versus grades 0–2. Within each severity stratum, LOS and charges decreased across increasing albumin categories (**Table 3**). Exact agreement for Wagner grade was 77.0% (314/408), with weighted κ = 0.58 (95% CI, 0.52–0.64). Exact agreement for IWGDF/IDSA infection grade was 82.4% (336/408), with weighted κ = 0.64 (95% CI, 0.57–0.71).

**Table 2:** Key indicators and outcomes by Wagner grade and IWGDF/IDSA infection grade.

Variable	Wagner 0–3 (*n* = 317)	Wagner 4–5 (*n* = 91)	*p*-value	Infection 0–2 (*n* = 351)	Infection 3 (*n* = 57)	*p*-value
HbA1c, %	7.9 ± 1.7	8.1 ± 1.9	0.315	7.9 ± 1.7	8.3 ± 1.7	0.103
Serum albumin, g/L	36.6 ± 5.7	33.7 ± 6.0	<0.001	36.6 ± 5.6	31.9 ± 5.6	<0.001
WBC, ×10^9^/L	8.3 ± 2.5	8.6 ± 2.5	0.357	8.3 ± 2.5	8.6 ± 2.5	0.381
CRP, mg/L	14 (8–26)	17 (10–25)	0.141	14 (8–26)	17 (9–26)	0.401
LOS, days	13 (8–18)	17 (10–25)	<0.001	13 (8–19)	16 (13–25)	<0.001
Nominal hospital charges, CNY	16,886 (11,629–24,547)	26,156 (18,122–38,478)	<0.001	18,036 (12,189–26,435)	23,337 (16,886–36,108)	<0.001

Values are mean ± SD for HbA1c, serum albumin, and WBC and median (IQR) for CRP, LOS, and charges. *p*-values are from Welch *t*-tests or Mann–Whitney tests, as appropriate. CRP, C-reactive protein; HbA1c, glycated hemoglobin; LOS, length of stay; WBC, white blood cell count.

**Table 3:** Length of stay and nominal hospital charges by albumin stratum within disease-severity categories.

Disease-severity stratum	Albumin stratum	LOS, days, median (IQR)	Charges, CNY, median (IQR)
Wagner 0–3	<30 g/L (*n* = 36)	20 (14–26)	25,034 (16,804–33,760)
	30 to <35 g/L (*n* = 73)	14 (11–21)	19,844 (14,998–30,787)
	≥35 g/L (*n* = 208)	12 (8–15)	14,436 (10,402–21,960)
Wagner 4–5	<30 g/L (*n* = 20)	24 (18–27)	42,577 (27,804–59,141)
	30 to <35 g/L (*n* = 28)	18 (14–25)	26,123 (18,294–39,214)
	≥35 g/L (*n* = 43)	13 (8–24)	20,783 (16,573–30,126)
Infection 0–2	<30 g/L (*n* = 36)	20 (16–24)	29,540 (21,198–38,471)
	30 to <35 g/L (*n* = 79)	15 (9–22)	21,591 (15,324–32,026)
	≥35 g/L (*n* = 236)	12 (8–16)	15,435 (10,843–23,308)
Infection 3	<30 g/L (*n* = 20)	24 (17–33)	27,785 (22,446–59,323)
	30 to <35 g/L (*n* = 22)	16 (14–24)	26,358 (18,074–37,906)
	≥35 g/L (*n* = 15)	13 (8–14)	15,361 (12,450–21,590)

Values are descriptive medians (IQRs). LOS, length of stay.

### 3.5 Adjusted associations with hospitalization burden

After management-extended multivariable adjustment, lower albumin remained associated with both outcomes (**Table 4**, **Figure 3**). Relative to albumin ≥35 g/L, albumin 30 to <35 g/L was associated with longer LOS (aRR, 1.24; 95% CI, 1.10–1.40) and higher charges (aCR, 1.28; 95% CI, 1.12–1.46). Albumin <30 g/L was associated with an aRR of 1.52 (95% CI, 1.29–1.78) and an aCR of 1.67 (95% CI, 1.40–1.99). When modeled continuously, each 5-g/L lower albumin was associated with a 19% longer expected LOS (aRR, 1.19; 95% CI, 1.14–1.25) and 22% higher expected charges (aCR, 1.22; 95% CI, 1.16–1.28). These estimates are management-conditional prognostic associations rather than causal effects.

**Table 4:** Management-extended and baseline-only adjusted associations between early-admission albumin and hospitalization burden.

Albumin contrast	Management-extended LOS aRR (95% CI)	Baseline-only LOS aRR (95% CI)	Management-extended charges aCR (95% CI)	Baseline-only charges aCR (95% CI)	Log-scale change, LOS/charges, %
30 to <35 vs. ≥35 g/L	1.24 (1.10–1.40)	1.25 (1.10–1.41)	1.28 (1.12–1.46)	1.28 (1.12–1.47)	2.97/2.56
<30 vs. ≥35 g/L	1.52 (1.29–1.78)	1.52 (1.29–1.78)	1.67 (1.40–1.99)	1.66 (1.39–1.99)	0.28/0.83
Per 5-g/L lower albumin	1.19 (1.14–1.25)	1.19 (1.14–1.25)	1.22 (1.16–1.28)	1.22 (1.16–1.28)	0.34/0.78

**Figure 3:**
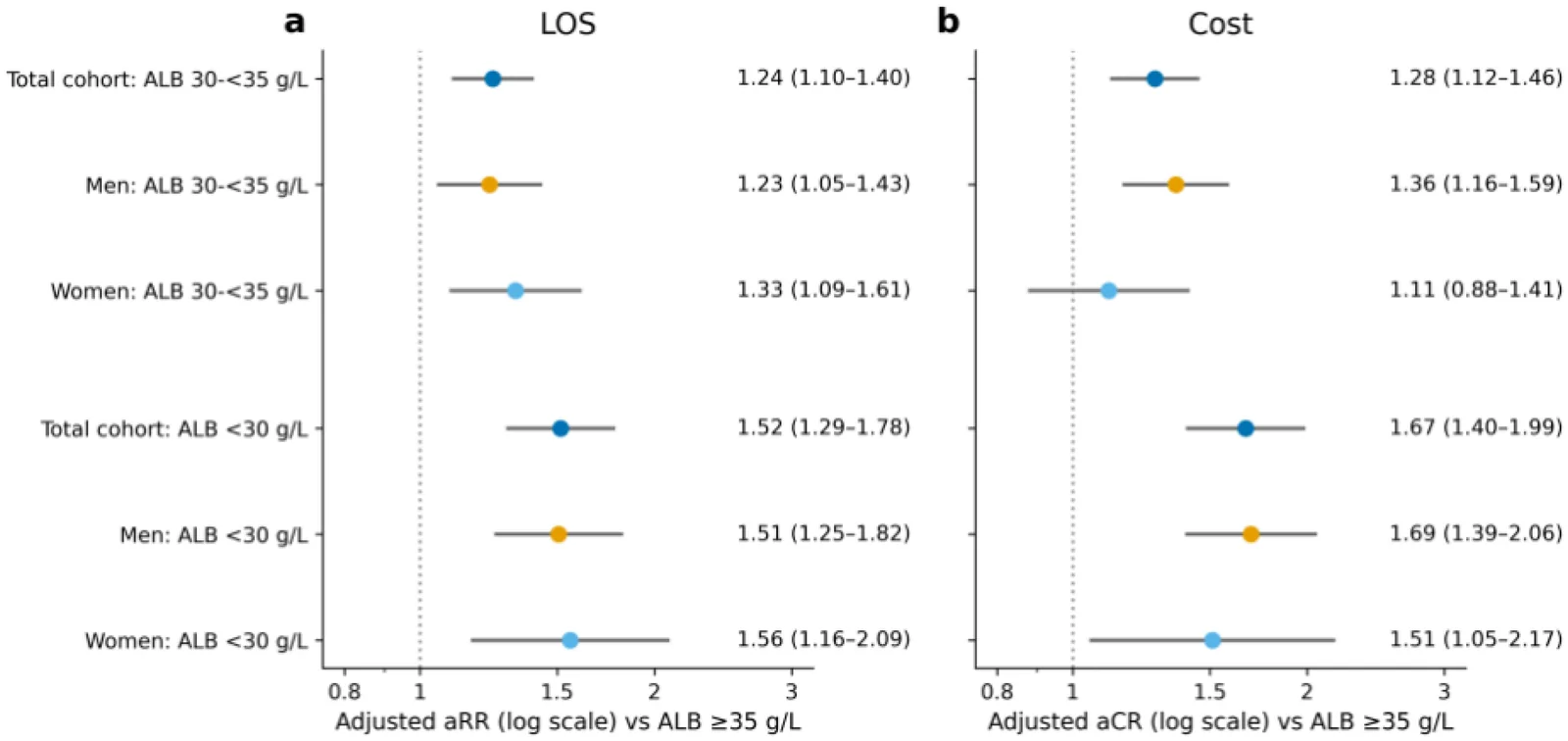
Adjusted associations of early-admission albumin categories with hospitalization burden relative to albumin ≥35 g/L. **(a)** LOS, expressed as adjusted rate ratios. **(b)** Nominal hospital charges, expressed as adjusted charge ratios. Estimates and 95% CIs are shown for the total cohort, men, and women. Sex-stratified estimates are descriptive; formal interaction tests did not identify effect modification. aCR, adjusted charge ratio; aRR, adjusted rate ratio; ALB, serum albumin; CI, confidence interval; LOS, length of stay.

Management-extended models included all baseline and management/subsequent-course covariates; baseline-only models excluded revascularization, negative-pressure wound therapy, antipseudomonal antimicrobial coverage, prior in-hospital antibiotics, and MDR PA phenotype. Categorical-albumin models used factor coding for Wagner grade, IWGDF/IDSA grade, smoking status, and admission year; continuous-albumin models used ordered grade scores and a centered linear admission-year term. Percentage changes were calculated on the log-ratio scale using unrounded model coefficients. All displayed albumin estimates had *p* < 0.001. aCR, adjusted charge ratio; aRR, adjusted rate ratio; CI, confidence interval; LOS, length of stay; MDR, multidrug resistant; PA, *Pseudomonas aeruginosa*.

### 3.6 Sensitivity analyses, multicollinearity, and influence diagnostics

Excluding the five management or subsequently ascertained variables produced nearly identical estimates (**Table 4**). For categorical contrasts, log-scale changes between baseline-only and management-extended models ranged from 0.28% to 2.97% for LOS and from 0.83% to 2.56% for charges. For each 5-g/L lower albumin, the baseline-only aRR was 1.19 (95% CI, 1.14–1.25) and the baseline-only aCR was 1.22 (95% CI, 1.16–1.28), corresponding to log-scale changes of 0.34% and 0.78%, respectively. Thus, the reported association was not materially dependent on adjustment for the recorded management variables, although unmeasured confounding and mediation remain possible.

There was no evidence of problematic multicollinearity. In continuous models, VIFs were 1.16 for Wagner grade and 1.21 for IWGDF/IDSA infection grade, and the maximum VIF was 1.39. In categorical models, adjusted GVIFs for Wagner grade were 1.05 for LOS and 1.04 for charges; corresponding values for IWGDF/IDSA grade were 1.08 and 1.07. Standardizing nominal charges to 2018 CNY did not materially change the continuous albumin estimate (nominal aCR, 1.219; 95% CI, 1.160–1.282; 2018-CNY aCR, 1.220; 95% CI, 1.160–1.282); categorical estimates were unchanged to the displayed precision because year-specific multiplicative factors were absorbed by the admission-year indicators. No observation had Cook’s distance >1. Thirty observations in the LOS model and 31 in the charge model exceeded the absolute albumin DFBETA threshold of 2/√*N*; after their exclusion, estimates remained directionally consistent (LOS aRR, 1.21; 95% CI, 1.16–1.27; charges aCR, 1.24; 95% CI, 1.18–1.30).

Adding FPG changed the albumin effect estimate by less than 0.2% on the log-ratio scale; FPG was not independently associated with LOS (*p* = 0.424) or charges (*p* = 0.708). In the post-PA LOS sensitivity model, each 5-g/L lower albumin was associated with an aRR of 1.15 (95% CI, 1.09–1.22). These analyses support robustness of the within-cohort association but do not eliminate selection caused by the serial-culture eligibility criterion.

### 3.7 Dose–response pattern and interaction by sex

Restricted cubic spline analyses showed monotonic decreases in predicted LOS and charges with increasing albumin (**Figure 4**). There was no statistical evidence of departure from linearity (*p* = 0.94 for LOS and *p* = 0.99 for charges) or albumin-by-sex interaction (*p* = 0.18 and *p* = 0.92, respectively). Thus, the analyses provided no evidence of nonlinearity, a sex-specific effect, or a biological inflection point within the observed range.

**Figure 4:**
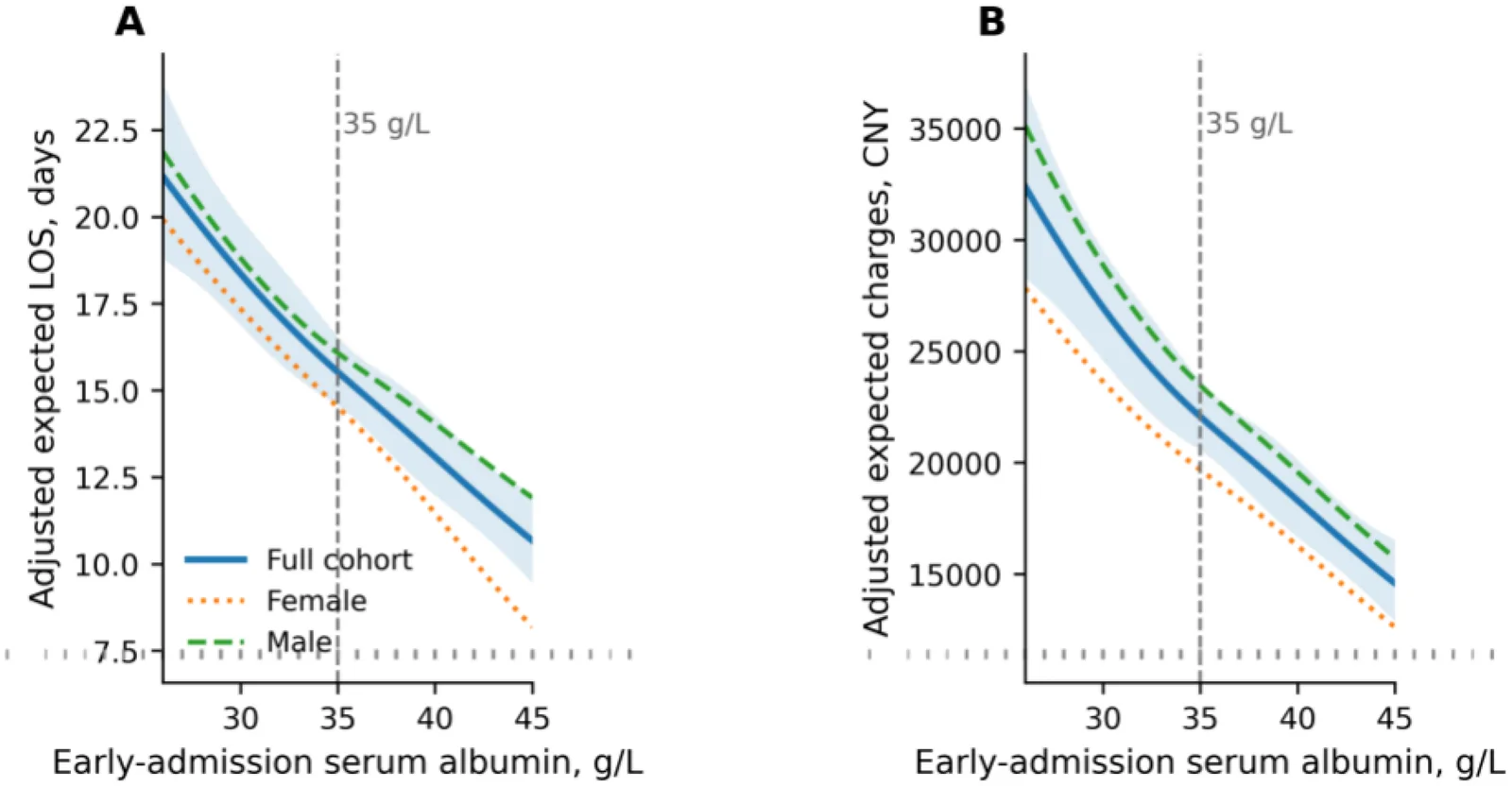
Adjusted dose–response associations of early-admission albumin with expected LOS **(a)** and nominal hospital charges **(b)**. Restricted cubic spline models used knots at the 5th, 35th, 65th, and 95th percentiles of albumin. Solid blue curves show full-cohort predictions with 95% confidence bands; dotted and dashed curves show descriptive sex-specific predictions, and rug marks show the observed albumin distribution. The vertical dashed line marks 35 g/L for orientation only. There was no evidence of nonlinearity or albumin-by-sex interaction. ALB, serum albumin; CNY, Chinese yuan; LOS, length of stay.

### 3.8 Exploratory operational threshold

For the exploratory high-burden endpoint, 153 of 408 patients met the full-cohort sex-specific 75th-percentile definition. The Youden-optimal threshold was 35 g/L, with an apparent AUC of 0.746 (95% CI, 0.696–0.791), sensitivity of 69.3%, and specificity of 67.8% (**Supplementary Figure 1**). Across 2,000 bootstrap resamples, the median optimal cutoff was 35 g/L and the 95% bootstrap percentile interval was 33–37 g/L; exactly 35 g/L was selected in 52.4% of resamples. These results demonstrate sample-dependent threshold variability and identify only a candidate range for independent evaluation, not a biological or actionable cut-point.

### 3.9 Incremental prognostic value and same-center temporal assessment

In the full cohort, the core clinical model had an apparent AUC of 0.679, which increased to 0.758 after adding albumin (ΔAUC, 0.079; bootstrap 95% CI, 0.036–0.120; **Figure 5a**). Optimism-corrected AUCs were 0.643 and 0.729, respectively. The apparent Brier score improved from 0.210 to 0.188 (optimism-corrected, 0.222 to 0.200), and the Akaike information criterion decreased from 519.9 to 477.4.

**Figure 5:**
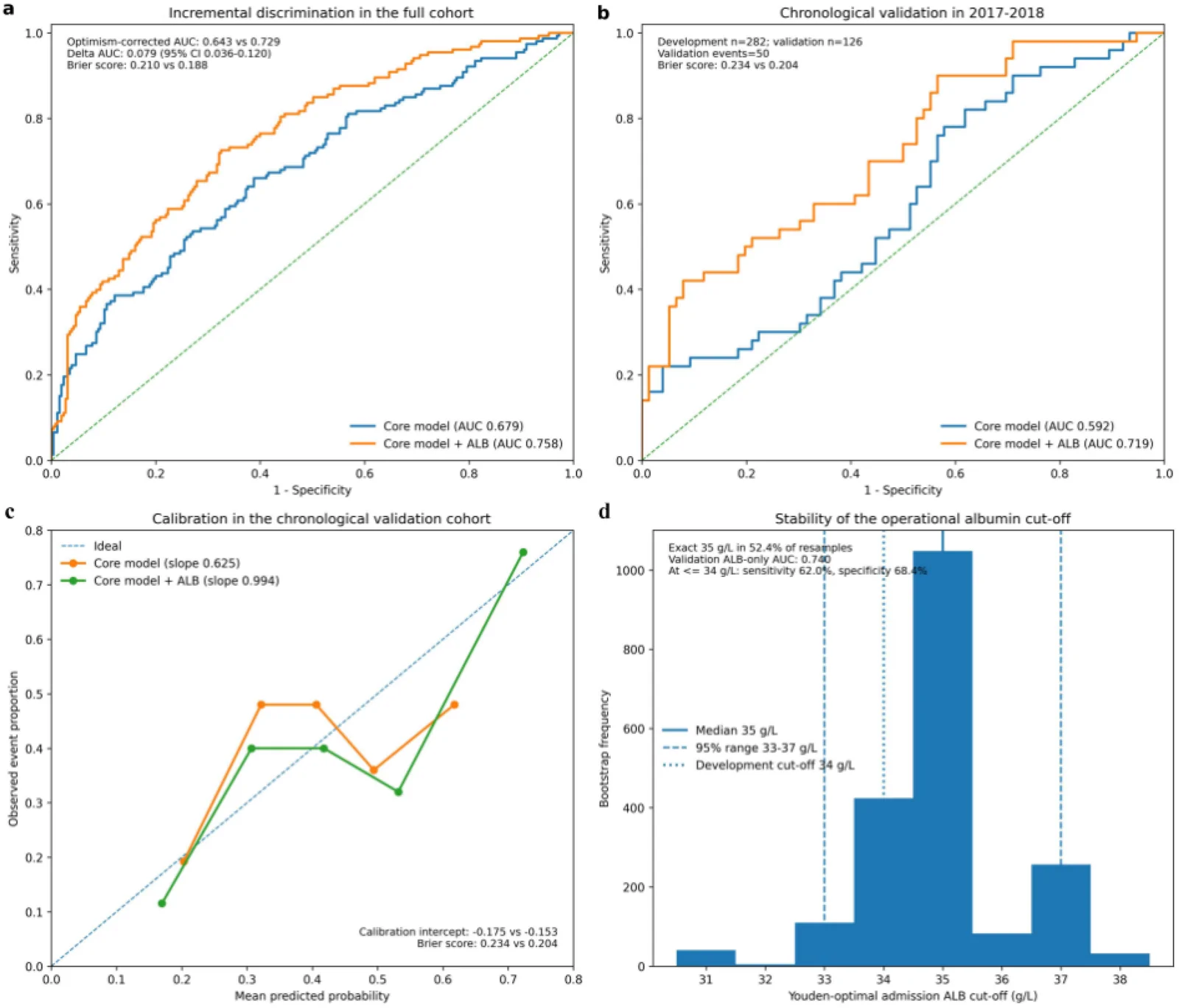
Exploratory incremental prognostic value, same-center temporal assessment, and cutoff stability. **(a)** ROC curves for the core model with and without albumin in the full cohort. **(b)** ROC curves in the 2017–2018 temporal assessment sample after development in 2013–2016. **(c)** Grouped calibration in the assessment sample. **(d)** Distribution of the Youden-optimal albumin cutoff across 2,000 bootstrap resamples. The 34-g/L development value was estimated in the smaller 2013–2016 subset, whereas 35 g/L was the full-cohort optimum; both are data-derived and hypothesis-generating. The assessment sample included 126 patients and 50 events, so performance estimates are descriptive, potentially imprecise, and require geographically independent external validation. ALB, serum albumin; AUC, area under the curve; ROC, receiver operating characteristic.

The chronological development sample included 282 patients, and the 2017–2018 assessment sample included 126 patients, of whom 50 met the high-burden endpoint defined using the frozen development-period thresholds. Assessment AUC increased from 0.592 for the core model to 0.719 after adding albumin, and the Brier score decreased from 0.234 to 0.204 (**Figure 5b**). The calibration slope changed from 0.625 to 0.994, with calibration intercepts of −0.175 and −0.153, respectively (**Figure 5c**). Because only 50 events were available in the assessment sample, these slope and discrimination estimates are imprecise; the apparent improvement may reflect sampling variation or overfitting and should not be interpreted as evidence that albumin corrected overfitting or established transportability. For continuous outcomes, adding albumin reduced assessment MAE from 5.91 to 5.57 days and RMSE from 7.93 to 7.16 days for LOS; corresponding charge errors decreased from CNY 9,259 to CNY 8,381 for MAE and from CNY 12,785 to CNY 11,975 for RMSE.

The albumin-only cutoff selected in the 2013–2016 development period was 34 g/L. In the 2017–2018 assessment cohort, continuous albumin alone yielded an AUC of 0.740. Applying the prespecified 34-g/L cutoff gave a sensitivity of 62.0% and a specificity of 68.4%. The 34-g/L development value differs from the 35-g/L full-cohort optimum because it was estimated in a smaller development subset using development-period outcome thresholds that were frozen before assessment. This expected variation under data-driven cut-point optimization reinforces that neither value is a definitive threshold (**Figure 5**).

### 3.10 Robustness across clinical subgroups

The association per 5-g/L lower albumin was directionally consistent across sex, Wagner grade, infection grade, CRP category, MDR PA, prior antibiotic exposure, osteomyelitis, and admission period (**Supplementary Figure 2**). No interaction reached conventional statistical significance; the smallest interaction *p*-value was 0.059 for LOS and 0.100 for charges. These analyses were exploratory and had limited power to detect interaction.

## 4 Discussion

In this retrospective single-center cohort, lower early-admission albumin was associated with longer LOS and higher institution-specific nominal charges among patients meeting the serial-culture criterion. Baseline-only estimates changed by less than 3% on the log-ratio scale, and inflation-standardized and influence-sensitivity analyses yielded the same overall interpretation. The association persisted when LOS was counted from the first PA-positive culture (aRR per 5-g/L lower albumin, 1.15; 95% CI, 1.09–1.22). There was no evidence of nonlinearity across the observed range, and albumin added exploratory prognostic information in a later same-center sample. These findings identify albumin as an associated host-risk marker within this selected cohort, not as evidence of a causal, clinically actionable, or nutritionally reversible pathway.

The culture-timing data are central to interpretation. Repeat cultures were not performed as routine surveillance but were obtained mainly during documented clinical reassessment, including persistent or worsening local findings and peri-debridement or intraoperative evaluation. Patients also had to remain hospitalized long enough for a second specimen to be collected and PA to be detected. Consequently, the cohort is enriched for complex clinical courses and does not represent an unselected population of hospitalized patients with DFU.

The study did not test whether subsequent PA detection increased hospitalization burden relative to other microbiological trajectories. Instead, it evaluated whether baseline systemic condition, represented by early-admission albumin, was associated with variation in LOS and charges among patients who shared this serial-culture and reassessment pathway. The findings therefore support an integrated host–disease–microbiology–care interpretation rather than a pathogen-only explanation of resource use.

The findings are consistent with reports linking lower albumin and inflammation-nutrition indices to impaired DFU healing, amputation, and postoperative outcomes (16–21). The present study extends that literature by quantifying the continuous association in a serially defined PA-detection cohort and evaluating incremental information beyond routine clinical variables. The lack of evidence for nonlinearity supports reporting the continuous association while avoiding interpretation of 35 g/L as a biological boundary. However, ASPEN states that albumin should not be used as a nutrition marker, and GLIM requires at least one phenotypic and one etiologic criterion for malnutrition (14, 15). Kidney filtration and albuminuria are complementary rather than interchangeable dimensions of diabetic kidney disease (40). Although serum creatinine-derived eGFR was included in the multivariable models and mean eGFR did not differ across albumin strata, preserved eGFR does not exclude albuminuric diabetic kidney disease. Because paired urine albumin and urine creatinine measurements and timed urinary protein quantification were unavailable, renal protein loss remains an unmeasured potential confounder; eGFR adjustment cannot close this pathway. WBC and CRP were included, but they do not comprehensively capture inflammatory burden or its timing. Differential-count-based indices could not be derived, and a CRP-to-albumin ratio would not constitute independent confounder adjustment because it contains albumin itself. Accordingly, the findings represent an association after adjustment for measured covariates, not an effect independent of nephropathy, inflammation, malnutrition, or other unmeasured pathways.

The association is biologically plausible but cannot be interpreted as causal. Albumin is a negative acute-phase reactant and may decrease because of inflammation, capillary leakage, reduced hepatic synthesis, renal or wound protein loss, inadequate intake, or hemodilution (14, 21, 36). It may therefore index systemic illness, frailty, and impaired repair capacity rather than represent an isolated nutritional deficiency. PA biofilm formation and antimicrobial resistance are possible management considerations, but PA detection in this study was an organism-level marker and did not demonstrate a PA-mediated pathogenic mechanism (8, 37–39). A low early-admission albumin value should be interpreted in a clinical context and may, where otherwise indicated, prompt structured assessment of infection severity, perfusion, renal and hepatic disease, protein loss, fluid status, dietary intake, weight trajectory, muscle or frailty status, and discharge needs. It should not automatically trigger albumin infusion, nutritional supplementation, broader antipseudomonal therapy, or any other intervention without an independently established indication.

The unadjusted difference in WBC across albumin strata, despite no corresponding difference in CRP, does not exclude inflammatory confounding. These markers have different kinetics and determinants: WBC may change rapidly with physiological stress or medication exposure, whereas CRP may lag the clinical trigger or be attenuated after treatment. Because the timing of CRP measurement relative to antibiotics and other early treatment could not be reconstructed reliably, the discordance should not be interpreted as evidence that inflammation was unrelated to albumin.

The estimated charge association was essentially unchanged after general inflation standardization. Nevertheless, the outcome represents institution-specific billed charges rather than economic costs. National all-items inflation cannot fully account for local medical tariffs, treatment regimens, procedure mix, or institutional accounting practices; absolute monetary values should therefore not be generalized directly to other hospitals or contemporary practice.

The threshold analysis also requires caution. The full-cohort optimum was 35 g/L, the development sample selected 34 g/L, and the bootstrap 95% range was 33–37 g/L. This variation reflects sample-dependent optimization and supports, at most, a candidate range for external testing rather than a universal cut-point. Decision-curve analysis is meaningful only when the decision, benefit, and harm attached to a threshold are explicit (34, 35). No albumin-triggered care pathway was tested; the proposed range should therefore guide future validation studies rather than mandate albumin infusion, nutrition therapy, surgery, revascularization, or broader antipseudomonal treatment.

The prognostic analyses were exploratory. Adding albumin was associated with numerically better discrimination, overall prediction error, and calibration point estimates in the full cohort and later temporal sample. However, the development and assessment samples came from the same center, laboratory, electronic record system, and clinical service; this was a same-center temporal assessment within the source system, not geographically or institutionally external validation. The assessment sample contained only 50 events relative to the model complexity, so the change in calibration slope from 0.625 to 0.994 may reflect sampling variation or overfitting and should not be read as evidence of generalizability. Performance also remains sensitive to case mix, the data-derived high-burden definition, local accounting, and care pathways (23–26). The model is not clinically ready; the findings show exploratory incremental prognostic information, not a basis for treatment decisions or proof that albumin independently determines hospitalization burden.

Several limitations materially constrain interpretation. First, eligibility depended on a later PA-positive culture. Patients had to survive and remain hospitalized long enough for clinically triggered repeat sampling and PA detection; conditioning on this post-admission event and differential time at risk creates immortal-time and collider selection that conventional covariate adjustment cannot remove (13). Although the association remained directionally similar for post-PA LOS, that analysis still conditions on continued hospitalization and repeat sampling; no analogous time-segmented charge analysis was reported, and total charges include expenditures accrued before cohort ascertainment. Second, repeat-culture indications and sampling techniques were retrospectively abstracted and may be misclassified. More than half of PA-positive specimens were wound-secretion or superficial samples; colonization, differences in sampling depth, specimen quality, and initial false-negative recovery therefore remain plausible explanations for the serial pattern. Culture positivity cannot prove nosocomial acquisition or a PA-mediated mechanism.

Additional limitations include residual confounding inherent to the retrospective design; potential early hemodilution because 26.5% of patients received crystalloid before albumin measurement; only moderate-to-substantial inter-rater agreement for retrospective severity grading; absence of paired urine albumin and urine creatinine measurements and 24-h urinary protein quantification, leaving albuminuric kidney disease and renal protein loss unmeasured despite eGFR adjustment; incomplete differential leukocyte and platelet data, precluding more comprehensive inflammatory indices; absence of validated nutritional, frailty, hepatic synthetic-function, and fluid-status assessments; and potential unmeasured mediation by treatment intensity. The high-burden endpoint was data-derived and sex-specific, the same-center temporal assessment sample was modest relative to model complexity, and no albumin-triggered pathway was evaluated. Charges were institution-specific and reflected nonstandardized treatment regimens and procedure mix; the 2013–2018 cohort may not reflect current multidisciplinary DFU, antimicrobial-stewardship, vascular, and wound-care practices. Healing, amputation, readmission, recurrence, limb salvage, epithelialization, and mortality were not available. These limitations restrict causal interpretation, prognostic transportability, and contemporary generalizability.

Given the relatively small sample size of the present study, the observed associations, particularly those involving albumin, should be regarded as exploratory and hypothesis-generating rather than as evidence of causality. Future studies should begin with a sufficiently powered, unselected inception cohort of patients admitted with DFU and prospectively document the timing, indication, sampling depth, and clinical interpretation of every culture. The cohort should distinguish initial detection of PA, subsequent PA detection, persistently non-PA cultures, and absence of repeat sampling; capture antimicrobial exposure; obtain GLIM-compatible measures of nutritional status and frailty; characterize protein loss and fluid status; and adjudicate clinical infection using prespecified criteria. Associations between continuously measured albumin and clinical outcomes, together with the incremental predictive performance attributable to albumin, should be evaluated in geographically independent, contemporary cohorts with adequate sample sizes and external validation. Any investigation of causality will require substantially larger, longitudinally characterized populations, rigorous control of confounding, and complementary causal-inference approaches, including Mendelian randomization when valid genetic instruments are available (41, 42) and its core assumptions are defensible. Only after external validation should a prespecified albumin-informed assessment pathway be prospectively evaluated against patient-centered outcomes and resource use.

## 5 Conclusions

Among hospitalized patients with DFU who met the serial-culture criterion of subsequent PA detection after an initial non-PA wound culture, lower early-admission albumin was associated with longer LOS and higher institution-specific hospital charges after adjustment for measured covariates. This wording should not be interpreted as an effect independent of unmeasured albuminuria, renal protein loss, or inflammatory burden. Similar estimates in baseline-only and other sensitivity analyses support the stability of this within-cohort association but do not resolve selection bias, residual confounding, or outcome-time circularity. The exploratory prognostic and threshold findings require geographically independent, contemporary validation and evaluation within a prespecified clinical pathway before any clinical use. These results do not establish causality, diagnose malnutrition, identify an albumin-replacement target, or generalize to all hospitalized patients with DFU.

## Data Availability

The raw data supporting the conclusions of this article will be made available by the authors, without undue reservation.

## Supplementary material

Supplementary Figure 1 Exploratory discrimination, calibration, and decision-curve analyses for the internally derived albumin threshold. **(A)** ROC curves in the full cohort and by sex. **(B)** Decision-curve analysis compared with treat-all and treat-none strategies. **(C)** Apparent calibration plot and Brier score. No albumin-triggered intervention was evaluated; therefore, the threshold and net-benefit findings are hypothesis-generating rather than evidence of clinical utility. ALB, serum albumin; AUC, area under the ROC curve; ROC, receiver operating characteristic.

Supplementary Figure 2 Subgroup robustness of the continuous albumin association. Adjusted associations per 5-g/L lower early-admission albumin are shown for **(A)** LOS and **(B)** nominal hospital charges. Interaction P values test heterogeneity. These analyses were exploratory and had limited power to detect interaction. aCR, adjusted charge ratio; aRR, adjusted rate ratio; ALB, serum albumin; LOS, length of stay; MDR, multidrug resistant; PA, *Pseudomonas aeruginosa*.

## References

[1] Zhang P Lu J Jing Y Tang S Zhu D Bi Y . Global epidemiology of diabetic foot ulceration: a systematic review and meta-analysis. Ann Med. (2017) 49:106–16. doi: 10.1080/07853890.2016.1231932 27585063

[2] McDermott K Fang M Boulton AJM Selvin E Hicks CW . Etiology, epidemiology, and disparities in the burden of diabetic foot ulcers. Diabetes Care. (2023) 46:209–21. doi: 10.2337/dci22-0043 36548709 PMC9797649

[3] Armstrong DG Tan TW Boulton AJM Bus SA . Diabetic foot ulcers: a review. JAMA. (2023) 330:62–75. doi: 10.1001/jama.2023.10578 37395769 PMC10723802

[4] Dickson MC Skrepnek GH . Hospitalization and health resource utilization in emergency department cases of diabetic foot infections in the United States from 2012 to 2021. J Clin Med. (2024) 13:5361. doi: 10.3390/jcm13185361 39336851 PMC11432337

[5] Senneville E Albalawi Z van Asten S.A Abbas ZG Allison G Aragón-Sánchez J . IWGDF/IDSA guidelines on the diagnosis and treatment of diabetes-related foot infections (IWGDF/IDSA 2023). Diabetes Metab Res Rev. (2024) 40:e3687. doi: 10.1002/dmrr.3687 37779323

[6] Schaper NC van Netten JJ Apelqvist J Bus SA Fitridge R Game F . Practical guidelines on the prevention and management of diabetes-related foot disease (IWGDF 2023 update). Diabetes Metab Res Rev. (2024) 40:e3657. doi: 10.1002/dmrr.3657 37243927

[7] Monteiro-Soares M Hamilton EJ Russell DA Srisawasdi G Boyko EJ Mills JL . Guidelines on the classification of foot ulcers in people with diabetes (IWGDF 2023 update). Diabetes Metab Res Rev. (2024) 40:e3648. doi: 10.1002/dmrr.3648 37179483

[8] Garousi M Monazami Tabar S Mirazi H Farrokhi Z Khaledi A Shakerimoghaddam A . Epidemiology of Pseudomonas aeruginosa in diabetic foot infections: a global systematic review and meta-analysis. Germs. (2023) 13:362–72. doi: 10.18683/germs.2023.1406 38361543 PMC10866166

[9] Du F Ma J Gong H Bista R Zha P Ren Y . Microbial infection and antibiotic susceptibility of diabetic foot ulcer in China: a literature review. Front Endocrinol (Lausanne). (2022) 13:881659. doi: 10.3389/fendo.2022.881659 35663325 PMC9161694

[10] Veve MP Mercuro NJ Sangiovanni RJ Santarossa M Patel N . Prevalence and predictors of Pseudomonas aeruginosa among hospitalized patients with diabetic foot infections. Open Forum Infect Dis. (2022) 9:ofac297. doi: 10.1093/ofid/ofac297 PMC9301575 35873292

[11] Uçkay I Lebowitz D Kressmann B von Dach E Lipsky BA Gariani K . Pseudomonal diabetic foot infections: vive la différence? Mayo Clin Proc Innov Qual Outcomes. (2022) 6:250–6. doi: 10.1016/j.mayocpiqo.2022.04.005 PMC9130228 35634135

[12] McCreery RJ Lyden E Anderson M Van Schooneveld TC . Impact of a syndrome-specific antibiotic stewardship intervention on antipseudomonal antibiotic use in inpatient diabetic foot infection management. Antimicrob Steward Healthc Epidemiol. (2023) 3:e39. doi: 10.1017/ash.2023.123 36960089 PMC10028944

[13] Yadav K Lewis RJ . Immortal time bias in observational studies. JAMA. (2021) 325:686–7. doi: 10.1001/jama.2020.9151 33591334

[14] Evans DC Corkins MR Malone A Miller S Mogensen KM Guenter P . The use of visceral proteins as nutrition markers: an ASPEN position paper. Nutr Clin Pract. (2021) 36:22–8. doi: 10.1002/ncp.10588 33125793

[15] Cederholm T Jensen GL Correia MITD Gonzalez MC Fukushima R Higashiguchi T . GLIM criteria for the diagnosis of malnutrition: a consensus report from the global clinical nutrition community. Clin Nutr. (2019) 38:1–9. doi: 10.1016/j.clnu.2018.08.002 30181091

[16] Vlad LG Grosser JA Dodenhoff KA Peoples AE Aguilo-Seara G Molnar JA . Examining albumin as a bioindicator of healing capability in patients with diabetic foot ulcers: a retrospective review. Wounds. (2023) 35:E193–6. doi: 10.25270/wnds/23012 37347595

[17] Cheng P Dong Y Hu Z Huang S Cao X Wang P . Biomarker prediction of postoperative healing of diabetic foot ulcers: a retrospective observational study of serum albumin. J Wound Ostomy Continence Nurs. (2021) 48:339–44. doi: 10.1097/won.0000000000000780 34186553

[18] Coskun B Ayhan M Ulusoy S . Relationship between prognostic nutritional index and amputation in patients with diabetic foot ulcer. Diagnostics (Basel). (2024) 14:738. doi: 10.3390/diagnostics14070738 PMC11011454 38611651

[19] Lee SH Kim SH Kim KB Kim HS Lee YK . Factors influencing wound healing in diabetic foot patients. Med (Kaunas). (2024) 60:723. doi: 10.3390/medicina60050723 38792906 PMC11122953

[20] Lin C Liu J Sun H . Risk factors for lower extremity amputation in patients with diabetic foot ulcers: a meta-analysis. PloS One. (2020) 15:e0239236. doi: 10.1371/journal.pone.0239236 32936828 PMC7494323

[21] Gremese E Bruno D Varriano V Perniola S Petricca L Ferraccioli G . Serum albumin levels: a biomarker to be repurposed in different disease settings in clinical practice. J Clin Med. (2023) 12:6017. doi: 10.3390/jcm12186017 37762957 PMC10532125

[22] Schuster NA Rijnhart JJM Twisk JWR Heymans MW . Modeling non-linear relationships in epidemiological data: the application and interpretation of spline models. Front Epidemiol. (2022) 2:975380. doi: 10.3389/fepid.2022.975380 38455295 PMC10910897

[23] Collins GS Reitsma JB Altman DG Moons KGM . Transparent reporting of a multivariable prediction model for individual prognosis or diagnosis (TRIPOD): the TRIPOD statement. Ann Intern Med. (2015) 162:55–63. doi: 10.7326/M14-0697 25560714

[24] Steyerberg EW Harrell FE Jr . Prediction models need appropriate internal, internal–external, and external validation. J Clin Epidemiol. (2016) 69:245–7. doi: 10.1016/j.jclinepi.2015.04.005 25981519 PMC5578404

[25] Van Calster B McLernon DJ van Smeden M Wynants L Steyerberg EW . Calibration: the Achilles heel of predictive analytics. BMC Med. (2019) 17:230. doi: 10.1186/s12916-019-1466-7 31842878 PMC6912996

[26] Wolff RF Moons KGM Riley RD Whiting PF Westwood M Collins GS . PROBAST: a tool to assess the risk of bias and applicability of prediction model studies. Ann Intern Med. (2019) 170:51–8. doi: 10.7326/m18-1376 30596875

[27] von Elm E Altman DG Egger M Pocock SJ Gøtzsche PC Vandenbroucke JP . The Strengthening the Reporting of Observational Studies in Epidemiology (STROBE) statement: guidelines for reporting observational studies. Lancet. (2007) 370:1453–7. doi: 10.1016/S0140-6736(07)61602-X 18064739

[28] Thongprayoon C Cheungpasitporn W Mao MA Sakhuja A Kashani K . U-shape association of serum albumin level and acute kidney injury risk in hospitalized patients. PloS One. (2018) 13:e0199153. doi: 10.1371/journal.pone.0199153 29927987 PMC6013099

[29] Bravo-Molina A Linares-Palomino JP Vera-Arroyo B Salmerón-Febres LM Ros-Díe E . Inter-observer agreement of the Wagner, University of Texas and PEDIS classification systems for the diabetic foot syndrome. Foot Ankle Surg. (2018) 24:60–4. doi: 10.1016/j.fas.2016.10.009 29413776

[30] Jalilian M Shiri S . The reliability of the Wagner Scale for evaluation the diabetic wounds: a literature review. Diabetes Metab Syndr. (2022) 16:102369. doi: 10.1016/j.dsx.2021.102369 34929620

[31] McHugh ML . Interrater reliability: the kappa statistic. Biochem Med (Zagreb). (2012) 22:276–82. doi: 10.11613/bm.2012.031 PMC3900052 23092060

[32] Schisterman EF Cole SR Platt RW . Overadjustment bias and unnecessary adjustment in epidemiologic studies. Epidemiology. (2009) 20:488–95. doi: 10.1097/ede.0b013e3181a819a1 19525685 PMC2744485

[33] World Bank . Inflation, Consumer Prices (Annual %) - China. World Development Indicators. International Monetary Fund, International Financial Statistics Database. Available online at: https://data.worldbank.org/indicator/FP.CPI.TOTL.ZG?locations=CN (Accessed July 13, 2026).

[34] Vickers AJ van Calster B Steyerberg EW . A simple, step-by-step guide to interpreting decision curve analysis. Diagn Progn Res. (2019) 3:18. doi: 10.1186/s41512-019-0064-7 31592444 PMC6777022

[35] Vickers AJ Van Calster B Wynants L Steyerberg EW . Decision curve analysis: confidence intervals and hypothesis testing for net benefit. Diagn Progn Res. (2023) 7:11. doi: 10.1186/s41512-023-00148-y 37277840 PMC10243069

[36] Wiedermann CJ . Hypoalbuminemia as surrogate and culprit of infections. Int J Mol Sci. (2021) 22:4496. doi: 10.3390/ijms22094496 33925831 PMC8123513

[37] Afonso AC Oliveira D Saavedra MJ Borges A Simões M . Biofilms in diabetic foot ulcers: impact, risk factors and control strategies. Int J Mol Sci. (2021) 22:8278. doi: 10.3390/ijms22158278 34361044 PMC8347492

[38] Darwitz BP Genito CJ Thurlow LR . Triple threat: how diabetes results in worsened bacterial infections. Infect Immun. (2024) 92:e00509-23. doi: 10.1128/iai.00509-23 38526063 PMC11385445

[39] Qin S Xiao W Zhou C Pu Q Deng X Lan L . Pseudomonas aeruginosa: pathogenesis, virulence factors, antibiotic resistance, and emerging therapeutics. Signal Transduct Target Ther. (2022) 7:199. doi: 10.1038/s41392-022-01056-1 35752612 PMC9233671

[40] Kidney Disease: Improving Global Outcomes (KDIGO) CKD Work Group . KDIGO 2024 clinical practice guideline for the evaluation and management of chronic kidney disease. Kidney Int. (2024) 105:S117–314. doi: 10.1016/j.kint.2023.10.018 38490803

[41] Tan J Wang J Yang Y Zhu J Wang L Xu W . Causal association between gut microbiota composition and the risk of atrial fibrillation. J Transl Int Med. (2025) 13:241–52. doi: 10.1515/jtim-2025-0031 40896293 PMC12392079

[42] Tan J Xu W Hu S Wang J Wang L Zhu J . Genetic evidence linking lipoprotein(a) to cardiovascular disease and the potential role of aspirin: a Mendelian randomization study. Rev Cardiovasc Med. (2025) 26:39322. doi: 10.31083/rcm39322 41209110 PMC12593744

