# Supplementary material for: Early-admission serum albumin and hospitalization burden in a selected serial-culture cohort of patients with diabetic foot ulcers and subsequent Pseudomonas aeruginosa detection: a retrospective cohort study
Source: Front Endocrinol (Lausanne). 2026 Oct 1;17:1918069. doi: 10.3389/fendo.2026.1918069 (PMC13668586; doi:10.3389/fendo.2026.1918069)
Supplement: Supplementary file 1 [file SupplementaryFile1.docx]

# **Supplementary Figures**

**Supplementary Figure S1.** Exploratory discrimination, calibration, and decision-curve analyses for the internally derived albumin threshold. (A) ROC curves in the full cohort and by sex. (B) Decision-curve analysis compared with treat-all and treat-none strategies. (C) Apparent calibration plot and Brier score. No albumin-triggered intervention was evaluated; therefore, the threshold and net-benefit findings are hypothesis-generating rather than evidence of clinical utility. ALB, serum albumin; AUC, area under the ROC curve; ROC, receiver operating characteristic.

**Supplementary Figure S2.** Subgroup robustness of the continuous albumin association. Adjusted associations per 5-g/L lower early-admission albumin are shown for (A) LOS and (B) nominal hospital charges. Interaction P values test heterogeneity. These analyses were exploratory and had limited power to detect interaction. aCR, adjusted charge ratio; aRR, adjusted rate ratio; ALB, serum albumin; LOS, length of stay; MDR, multidrug resistant; PA, Pseudomonas aeruginosa.
